# Extraction of Biocompatible Collagen From Blue Shark Skins Through the Conventional Extraction Process Intensification Using Natural Deep Eutectic Solvents

**DOI:** 10.3389/fchem.2022.937036

**Published:** 2022-06-16

**Authors:** Miguel P. Batista, Naiara Fernández, Frédéric B. Gaspar, Maria do Rosário Bronze, Ana Rita C. Duarte

**Affiliations:** ^1^ iBET, Instituto de Biologia Experimental e Tecnológica, Oeiras, Portugal; ^2^ LAQV-REQUIMTE, Departamento de Química, Faculdade de Ciências e Tecnologia, Universidade Nova de Lisboa, Caparica, Portugal; ^3^ Instituto de Tecnologia Química e Biológica António Xavier, Universidade Nova de Lisboa, Oeiras, Portugal; ^4^ FFULisboa, Faculty of Pharmacy, University of Lisbon, Lisbon, Portugal

**Keywords:** extraction process intensification, marine waste valorization, natural deep eutectic solvent (NADES), blue sharkskin collagen, extract characterization

## Abstract

The disposal of large amounts of skin waste resulting from the blue shark fishing industry presents several industrial and environmental waste management concerns. In addition, these marine subproducts are interesting sources of collagen, a fibrous protein that shows high social and economic interest in a broad range of biomedical, pharmaceutical, and cosmetic applications. However, blue shark wasted skins are a poorly explored matrix for this purpose, and conventional collagen recovery methodologies involve several pre-treatment steps, long extraction times and low temperatures. This work presents a new green and sustainable collagen extraction approach using a natural deep eutectic solvent composed of citric acid:xylitol:water at a 1:1:10 molar ratio, and the chemical characterization of the extracted collagen by discontinuous electrophoresis, thermogravimetric analysis, Fourier transformed infrared spectroscopy and circular dichroism. The extracted material was a pure type I collagen, and the novel approach presented an extraction yield 2.5 times higher than the conventional one, without pre-treatment of raw material and reducing the procedure time from 96 to 1 h. Furthermore, the *in vitro* cytotoxicity evaluation, performed with a mouse fibroblasts cell line, has proven the biocompatibility of the extracted material. Overall, the obtained results demonstrate a simple, quick, cheap and environmentally sustainable process to obtain marine collagen with promising properties for biomedical and cosmetic applications.

## GRAPHICAL ABSTRACT

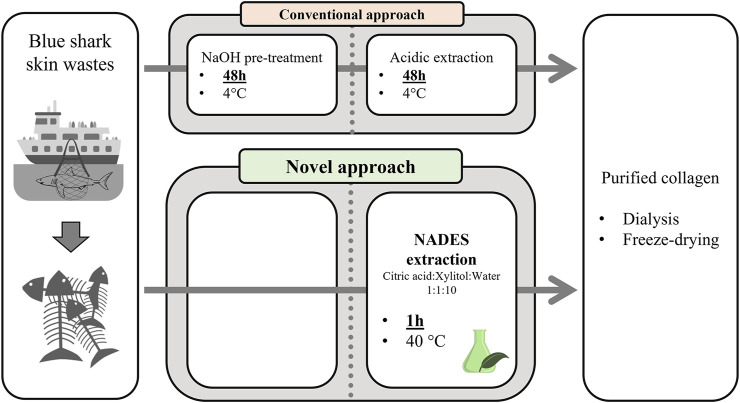

## 1 Introduction

Bioeconomic strategies in Europe involve turning organic waste and residues into valuable and safe bio-based products (European Commission, 2018). In EU-28, 88 Mt/year of food is wasted, with associated costs estimated at 143 B€ (Stenmarck et al., 2016). Among all wasted food, the fishing industries are responsible for discarding more than 50% of ocean fish tissues, including heads, skin, and viscera, representing a total of ca. 5.2 Mt/year in the EU (Caruso, 2015). The blue shark (*Prionace glauca*) is the most widely distributed and fished shark species globally. Its catch increased considerably in the late ‘90s for the consumption of shark meat, fillets, nutritional supplements, and fin soup (da Silva et al., 2021), leading to large amounts of shark skins being wasted. In the past, these residues were used for fertilizers or animal feed since they were considered of low value. However, several studies pointed out these marine by-products as excellent sources of high-added value biopolymers such as collagen (Jha and Kumar, 2019; Lionetto and Esposito Corcione, 2021; Mahmud et al., 2021). For instance, Vitorino & Filhos Lda (Peniche, Portugal), the raw material supplier for this study, is a good example, with a production of about 400 kg/day of blue shark skins as a result of industrial processing.

The fibrous collagen protein constitutes the primary structural element in the animal connective tissues (Shoulders and Raines, 2009). This protein consists of three parallel polypeptide *a*-chains forming a triple helix structure, giving origin to more than 29 types of collagen (Shoulders and Raines, 2009; Ricard-Blum, 2011). Besides collagen type I being the main structural element of human tissues’ extracellular matrix, collagen also has numerous intrinsic properties such as gelation capacity, biocompatibility, bioactivity, and biodegradability (Davison-Kotler et al., 2019; Lin et al., 2019; Coppola et al., 2020; Subhan et al., 2021). These aspects promote a high demand for this protein to develop products for the pharmaceutical, biomedical, cosmetic, and food industries. According to Markets and Markets™, the global collagen market is expected to grow in the following years, estimated to be valued at USD 4.1 billion in 2021 and is projected to reach USD 5.3 billion by 2026. Bovine and porcine are the most common collagen sources. Nonetheless, marine sources, such as fish biomass and by-catch organisms, have been rising in the last years as alternatives for obtaining this protein (Coppola et al., 2020; Subhan et al., 2021). However, the blue shark skin wastes are a matrix barely explored for this purpose.

The extraction of collagen for topical biomedical applications is a widely studied topic, and several publications address collagen extraction from marine sources using conventional methodologies and alternative solvents, such as deep eutectic solvents (DES) (Addad et al., 2011; Liu et al., 2015; Sotelo et al., 2016; Bai et al., 2017; Carvalho et al., 2018; Elango et al., 2018; Slimane and Sadok, 2018; Govindharaj et al., 2019; Jafari et al., 2020; Liu et al., 2020; Priyanka et al., 2020; Seixas et al., 2020; Bisht et al., 2021; Blidi et al., 2021). Conventional extractions involve several pre-treatment steps, long extraction times, and low temperatures (Addad et al., 2011; Liu et al., 2015; Sotelo et al., 2016; Carvalho et al., 2018; Elango et al., 2018; Slimane and Sadok, 2018; Govindharaj et al., 2019; Jafari et al., 2020; Liu et al., 2020; Priyanka et al., 2020; Seixas et al., 2020; Blidi et al., 2021). Therefore, alternative methods are needed to implement feasible industrial processes. DES are defined as mixtures of two or more components that present a high melting point depression at a particular composition, becoming liquids at room temperature (Hansen et al., 2021). This type of solvents obtained by the interaction between a hydrogen-bond acceptor and a hydrogen-bond donor has gained much relevance as a green and sustainable alternative to conventional industrial processes (Paiva et al., 2014; Hansen et al., 2021). These systems present several advantages, namely low price, a large number of different combinations, low toxicity, and biodegradability (Paiva et al., 2014; Hansen et al., 2021). More interest arises when the compounds that constitute the DES are primary metabolites, so-called natural DES or NADES. Representing all the green chemistry principles, the biocompatibility of NADES is of great interest when it comes to the pharmaceutical, medical, and food industries (Liu et al., 2018). Among the natural molecules, citric acid and xylitol have been widely used in forming NADES and have already been studied to extract phenolic and volatile aromatic compounds, metals, among others (Bajkacz and Adamek, 2017; Cunha and Fernandes, 2018; González et al., 2018; Aryati et al., 2020; Guinet et al., 2020; Oomen et al., 2020; Santana et al., 2020; Silva et al., 2020; Fanali et al., 2021; Osowska et al., 2021; Rodríguez-Juan et al., 2021). Although only a few published studies are related to their combined use, NADES composed of citric acid and xylitol, previously and extensively characterised by the works of Grønlien et al. and Guinet et al., present a promising potential for extracting biopolymers (Grønlien et al., 2020; Guinet et al., 2020). This is the case of the work by de Grønlien et al., who reported this NADES system as an adequate collagen solubilizing agent and as a potential excipient for collagen-based products (Grønlien et al., 2020).

This work aims to develop a new sustainable, faster, and more straightforward extraction of collagen with NADES composed of citric acid:xylitol:water to valorize currently undervalued blue shark skin wastes. The extract obtained by the proposed technology was compared to the one obtained using the conventional methodology in regard to protein purity and chemical characterization. Additionally, to evaluate the safety of the extracted material for potential topical applications, the *in vitro* cytotoxicity was evaluated on a mouse fibroblast cell line (NCTC clone 929 cells). This work is fully aligned with the circular economy concept by applying green and sustainable extraction techniques and promoting waste valorization through the isolation of a fibrous protein presenting a high social and economic interest with a low carbon footprint.

## 2 Materials and Methods

### 2.1 Materials

Blue shark (*Prionace glauca*) skins were removed from cold-stored fish body parts in an industrial plant and kindly provided by Vitorino & Filhos, Lda (Peniche, Portugal). Skins were washed with ice-cold deionized water and stored frozen at a temperature of −20°C until use. Acetic acid (99.7%) and citric acid were purchased from Panreac (Germany). Xylitol, commercial Type I collagen from calf skin and *ß*-mercaptoethanol were supplied by Sigma-Aldrich (United States). The protein ladder Precision Plus Protein™ All Blue Prestained Protein Standard, Laemmli Sample Buffer, Tris/Glycine/SDS Running Buffer, and Bio-Safe™ Coomassie Stain were purchased from Bio-Rad (United States). Minimum essential medium (MEM) with Earle′s balanced salts and 2.0 mM l-glutamine, phosphate-buffered saline (PBS), pH = 7.4, and dimethyl sulfoxide (DMSO) were purchased from Sigma (United States). Non-essential amino acids (NEAA), fetal bovine serum (FBS) and 0.25% (w/v) Trypsin-EDTA were purchased from Gibco (Life Technologies, United States). CellTiter 96® Aqueous Non-Radioactive Cell Proliferation Assay (MTS) reagent assay was obtained from Promega (Madison, United States).

### 2.2 NADES Preparation

NADES composed of citric acid:xylitol:water (molar ratio 1:1:10) were prepared according to the method presented by Grønlien (Grønlien et al., 2020) with some modifications. The three-component mixture was weighed into a round bottom flask, and the mixture was stirred in an oil bath at 50 ± 5°C until a homogenous and transparent liquid was obtained (∼2 h).

### 2.3 NADES Viscosity Measurement

The shear viscosity of NADES was measured using a rheometer (MCR 102, Anton Parr, Kinexus Pro+, Malvern) mounted with a parallel plate geometry (PP 50, Anton Parr) with a gap of 1 mm and a constant shear rate of 10 s^−1^. A temperature scan was performed from 4 to 55°C at 3°C/min. Viscosity results are expressed as a mean of three measurements.

### 2.4 Extraction of Collagen From Blue Shark Skin

Two extraction techniques were performed. First, acid-soluble collagen was extracted following the conventional extraction described in the literature (Carvalho et al., 2018). Then, after preliminary optimization tests, the methodology was adapted for collagen extraction using NADES, with some modifications. Frozen skins were freeze-dried for 48 h and milled (Retsch Cross Beater Hammer Mill Sk1, Germany) to obtain a powder. Next, the skin powder was mixed with NADES in a sample:solvent ratio of 1:10 (w:w) for 1 h at 40°C with continuous stirring. The solution was then centrifuged (Fisher Scientific Marathon 22KBR, United States) at 4500 rpm for 20 min. The supernatant was then dialyzed for 72 h against distilled water, with the solutions being changed every 12 h. The resulting extract was freeze-dried for 24 h and stored at room temperature until further use.

### 2.5 Global Extraction Yield and Total Protein Content of the Extract

The extraction yield was calculated using Eq. 1.

$$Yield\;\left(\%\right)=\frac{weight\;of\;dried\;extract}{weight\;of\;dried\;skins}\;X\;100$$ (1)

To determine the extract’s protein content, the obtained freeze-dried samples were dissolved in 0.5 M acetic acid at appropriate concentration and the total protein content was measured using the Lowry method, with bovine serum albumin as a standard (LOWRY et al., 1951).

### 2.6 Sodium Dodecyl Sulphate-Polyacrylamide Gel Electrophoresis (SDS-PAGE)

To evaluate the purity of the extracts and the molecular weight of the obtained protein fractions, a Bio-Rad Mini-PROTEAN® system (Bio-Rad, United States) was employed to separate proteins by SDS-PAGE. The lyophilized collagen samples were dissolved in 0.5 M acetic acid (2 mg/ml), mixed in a 1:1 (v:v) dilution with Laemmi sample buffer containing 5% (v/v) *ß*-mercaptoethanol and heated for 10 min at 70°C to denature the proteins. The 7.5% Mini-PROTEAN® TGX™ Precast Protein Gel (12-well, 20 μL) was loaded with 10 μL protein ladder and 10 μL from each collagen sample. The samples were run at 200 V for 30 min, and the gel was then stained using the Coomassie stain for 1 h with continuous stirring. Finally, the gel was detained in distilled water overnight and imaged using a 48-megapixel RGB camera.

### 2.7 Thermogravimetric Analysis (TGA)

To evaluate the thermal behavior profiles of the collagen extracts, TGA analysis was performed in a Thermal Analysis instrument (Labsys EVO, Setaram, Caluire, France), in an argon atmosphere, within a temperature range between 25 and 700°C and a 10°C/min heat ramp.

### 2.8 Fourier-Transform Infrared (FTIR) Spectroscopy

FTIR spectroscopy in attenuated total reflectance (ATR) mode was performed with a Thermo Scientific FTIR spectrometer (Class 1 Laser Product Nicolet 6100, San Jose, United States). The presence of collagen’s characteristic chemical bonds/groups was evaluated by recording 32 scans between 4,000–650 cm^−1^ with a resolution of 4 cm^−1^.

### 2.9 Protein Conformation

Circular dichroism (CD) spectra of extracts were recorded from 190 to 260 nm on a Chirascan™ qCD spectrometer/SX20 (Applied Photophysics, United Kingdom) using a 0.1 cm^−1^ path length cuvette. Dry collagen was dissolved at 1 mg/ml in 0.5 M acetic acid. Samples were loaded at 4°C into precooled CD cuvettes.

### 2.10 Hg Quantification

The presence of mercury in the blue shark skin and collagen extracts was outsourced to Silliker Portugal, S.A. Mercury was quantified by atomic absorption spectrophotometry on a DMA-80 EVO equipment (Milestone, Sorisole, Italy), with previous thermal decomposition of the samples and mercury amalgamation. Mercury quantification results are expressed as a mean of three measurements.

### 2.11 Biocompatibility

#### 2.11.1 Samples Preparation

The cytotoxicity of any leachables present in the collagen extracts that can migrate to the skin cells was evaluated following the methodology described in ISO 10993–5, a highly sensitive test used to determine medical devices’ toxicity (LI et al., 2015). The leaching of the samples was conducted according to the principles of test sample extraction described in the ISO guidelines. Specimens of each collagen extract were placed in glass vials in contact with the cell culture medium, used as the leaching medium, in a 0.1 g/ml extraction ratio. Sample vials were kept in a shaking water bath at 37 ± 1°C for 72 h. After extraction, the biocompatibility testing sample containing the extracted leachables were filter-sterilized using a 0.2 μm syringe filter and used for the cytotoxicity test.

#### 2.11.2 Cell Culture

Mouse fibroblasts NCTC clone 929 (ECACC 88102702) cells were purchased from the European Collection of Authenticated Cell Cultures (EACC, Public Health England, Salisbury, United Kingdom). Cells were routinely grown in a standard medium MEM supplemented with 1% (v/v) NEAA and 10% (v/v) heat-inactivated FBS. Stock cells were maintained as monolayers in 75 cm^2^ culture flasks, subcultured every week (seeding 30,000 cells/cm), and incubated at 37 °C in a 5% CO2 humidified atmosphere. For cell passage, the cells were detached when confluence reached about 80% using 0.25% (v/v) trypsin/EDTA at 37°C. The cells were collected, and viability was determined using the standard trypan blue staining procedure. Cell counting was performed using a hemocytometer. All cellular assays described below were performed with cells between passages 10 and 25.

#### 2.11.3 MTS Metabolic Activity Assay

Cell viability was quantified using the MTS cytotoxicity test, following the methodology described in the literature (Batista et al., 2020) with slight modifications. NCTC clone 929 cells were seeded into 96-well plates (100 μL volume) with a density of 1.0 × 104 cells/100 μL and maintained in culture for 24 h (∼1 doubling period) to form a semiconfluent monolayer. After 24 h, the culture media was replaced by 100 μL of the prepared biocompatibility testing sample and cells were incubated for 24 h. Lastly, the biocompatibility testing sample was removed, cells were rinsed with PBS and incubated for 2 h with 100 μL of MTS reagent assay, diluted according to the manufacturer’s information. The absorbance was recorded at 490 nm using a microplate spectrophotometer (EPOCH, 219 Bio- Tek, United States). Experiments were performed in triplicate in three independent assays. The positive control of cytotoxicity was done with a treatment of 10% (v/v) DMSO solution diluted in MEM.

Results were expressed as a percentage of cellular viability (Viab.%) relative to the control (untreated cells). A cytotoxic effect was considered for viability percentages below 70%, according to ISO 10993–5.

### 2.12 Statistical Analysis

Each experiment was performed at least in triplicate and all data are expressed as means ± standard errors (SD). The statistical analysis of the data was performed using GraphPad Prism 6 (GraphPad Software, Inc., CA). All values were tested for normal distribution and equal variance. When homogeneous variances were confirmed, data were analyzed by One Way Analysis of Variance (ANOVA) coupled with Tukey’s post hoc analysis to identify means with significant differences.

## 3 Results and Discussion

Despite some published works describing collagen extraction from marine sources using solid-liquid extractions with conventional solvents and DES, blue shark skin wastes have been barely explored for this purpose (Addad et al., 2011; Liu et al., 2015; Sotelo et al., 2016; Bai et al., 2017; Carvalho et al., 2018; Elango et al., 2018; Slimane and Sadok, 2018; Govindharaj et al., 2019; Jafari et al., 2020; Liu et al., 2020; Priyanka et al., 2020; Seixas et al., 2020; Bisht et al., 2021; Blidi et al., 2021). Due to their ability to solubilize collagen and biocompatible properties, NADES are interesting candidates as extraction solvents and potential excipients in collagen-based products. Recently, two studies have been published on collagen extraction from cod skins using NADES systems composed of choline chloride−oxalic acid and urea-lactic acid (Bai et al., 2017; Bisht et al., 2021). Although these systems are classified as NADES, choline salts and their esters (including choline chloride) and urea reaction products belong to the EU prohibited substances list in cosmetics (The European Parliament And The Council Of The European Union, 2009). This is a limitation for using these NADES as extraction solvents and product excipients of novel collagen-based products for potential applications in contact with the human skin. This work presents a new extraction methodology for recovering collagen from blue shark skins using bio-safe NADES. Based on the ability to dissolve collagen while maintaining its structural properties, the NADES system composed of citric acid:xylitol:water (molar ratio 1:1:10) was used to perform the extraction (Grønlien et al., 2020). Collagen was expected to be extracted through the protein diffusion to the liquid media due to the pH media decrease by the presence of citric acid. Xylitol was expected to act as a collagen thermostabilizing agent since polyols have been reported to stabilize the triple helix of collagen by binding to the protein surface and forming additional hydrogen bonds (Usha et al., 2006; Usha and Ramasami, 2008).

### 3.1 NADES Viscosity

**FIGURE 1:**
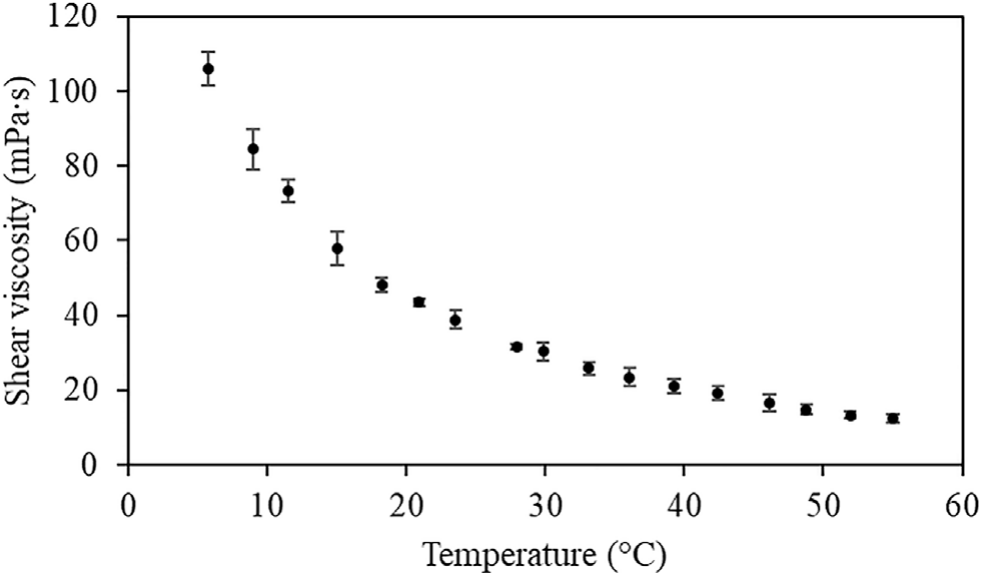
Variation of the shear viscosity of NADES system composed of citric acid:xylitol:water (molar ratio 1:1:10) as a function of temperature.

The inherent viscosity of most DES is an important factor in solid-liquid extractions as it regulates the mass transfer rate and influences the extraction yield. Although most DES exhibits relatively high viscosity, this property can be significantly controlled by temperature, mitigating the strength of intermolecular forces in DES, reducing their viscosity (Cunha and Fernandes, 2018; Liu et al., 2018). This physical characteristic is even more critical in this work since conventional collagen recovery methods are performed at low temperatures (about 4°C). Figure 1 presents the viscosity values as a function of temperature for the NADES system adapted in our new collagen extraction methodology.

A decrease in NADES viscosity with increasing temperature is observed. As expected, this NADES presented high viscosity (106 mPa s) when employing the low temperatures used in the conventional extraction methodology of collagen (4°C). A temperature increase impacts the intermolecular forces of DES and consequently decreases the solvent viscosity (Liu et al., 2018). Therefore, the extraction temperature is an important factor in regulating mass transfer rate and influencing the extraction yield. To ensure a low viscosity of the NADES system without affecting the collagen structural properties, the temperature of 40°C was chosen to carry out the new NADES extraction method. Extractions at higher temperatures (60 and 80°C) resulted in the hydrolysis of the collagen (Supplementary Figure S1.).

### 3.2 Global and Protein Extraction Yield of the Extracts and Collagen Purity

For the novel collagen extraction approach, freeze-dried skin powder was mixed with NADES in a sample:solvent ratio of 1:10 (w:w) for 1 h at 40°C. Collagen was also extracted following the conventional extraction methodology described in the literature (Carvalho et al., 2018). Both extracts were dialyzed, and the resulting acid-soluble collagen was freeze-dried. The global extraction yield, extracts’ total protein content and the protein extraction yield are reported in Table 1.

**TABLE 1:** Global extraction yield (%), extracts’ protein content (%) and the protein extraction yield (%) of the extracts obtained from blue shark skins with conventional solvent and NADES.

Methodology	Operating conditions		Extraction yield (%)		Protein content (%)
	NaOH 2016Pre-treatment	Collagen extraction	Global	Protein	Extract
Conventional	48 h; 4°C	48 h; 4°C	7.57 ± 3.53	6.69 ± 3.12	88.3 ± 4.94
NADES	—	1 h; 40°C	18.6 ± 3.82	16.1 ± 3.30	86.5 ± 10.9

The results showed a global extraction yield of 18.6% when collagen was recovered using NADES, 2.5 times higher than the yield obtained with conventional extraction (7.57%). Although blue shark skins are a barely explored matrix for collagen extraction, the results obtained with the conventional method agree with other works reported on the extraction of collagen from marine species skins (Jafari et al., 2020). In addition to the ability of citric acid:xylitol:water (molar ratio 1:1:10) NADES to dissolve collagen, this yield improvement may be related to the higher temperature (40°C) used in NADES extraction when compared to the conventional approach (4°C). As verified in Figure 1, the increasing temperature reduces the NADES viscosity, resulting in a higher mass transfer rate. The obtained results agree with the previous work of Bai et al., which showed that temperature has a positive influence on collagen extraction yield (Bai et al., 2017). Usually, collagen extraction involves extensive pre-treatment and extraction steps under low temperatures. However, the higher temperature combined with the chosen NADES system allowed to obtain a rich protein extract from shark skins, avoiding the raw material’s pre-treatment step and a noticeable shorter extraction time (1 h). The conventional methods involve complex acidic/basic treatments and multiple stages for the isolation and precipitation of collagen, which contributes to the low extraction yield.

**FIGURE 2:**
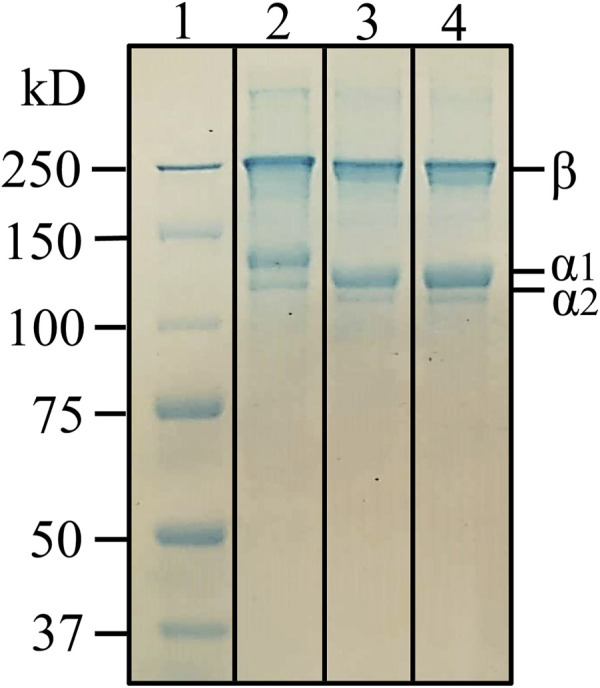
Electrophoretic profile of (1) molecular weight standards; (2) commercial collagen type I from calf skin; (3) protein rich extract using conventional solvent; (4) protein rich extract from NADES extraction.

Total protein content in the extracts was quantified using the Lowry method. The results showed that both extracts are rich in protein, above 87% of total protein content. Therefore, a superior protein yield using NADES methodology was obtained. The molecular weight of the obtained protein fractions was assessed by SDS-PAGE. Figure 2 shows SDS-PAGE band patterns of commercial calf skin collagen type I, used as standard, and the extracts obtained using NADES and the conventional methodologies.

Similar electrophoretic band patterns of the two obtained extracts with a commercial calf skin collagen standard were observed. This similarity of the bands’ distribution and molecular weights suggests that both extracts contain highly pure type I collagen, with a structure of two distinct *a*-chains of around 120 kDa and a beta component of about 200 kDa, a characteristic electrophoretic profile comparable with other fish species (Jafari et al., 2020). The slightly lower molecular weight of *a*-chains from the extracted collagens, when compared to the standard, may be due to the differences in the collagen animal source. This result agrees with the literature, as collagen from marine sources has been associated with differences in the amino acid composition compared to mammalian collagen (Carvalho et al., 2018). This characteristic makes collagen from marine sources more susceptible to high-temperature degradation and more susceptible to hydrolysis (Sotelo et al., 2016). However, although NADES extraction is performed at 40°C, the absence of bands below 100 kDa suggests that the selected NADES system stabilized and prevented collagen degradation and that the extracted collagen has high purity.

**FIGURE 3:**
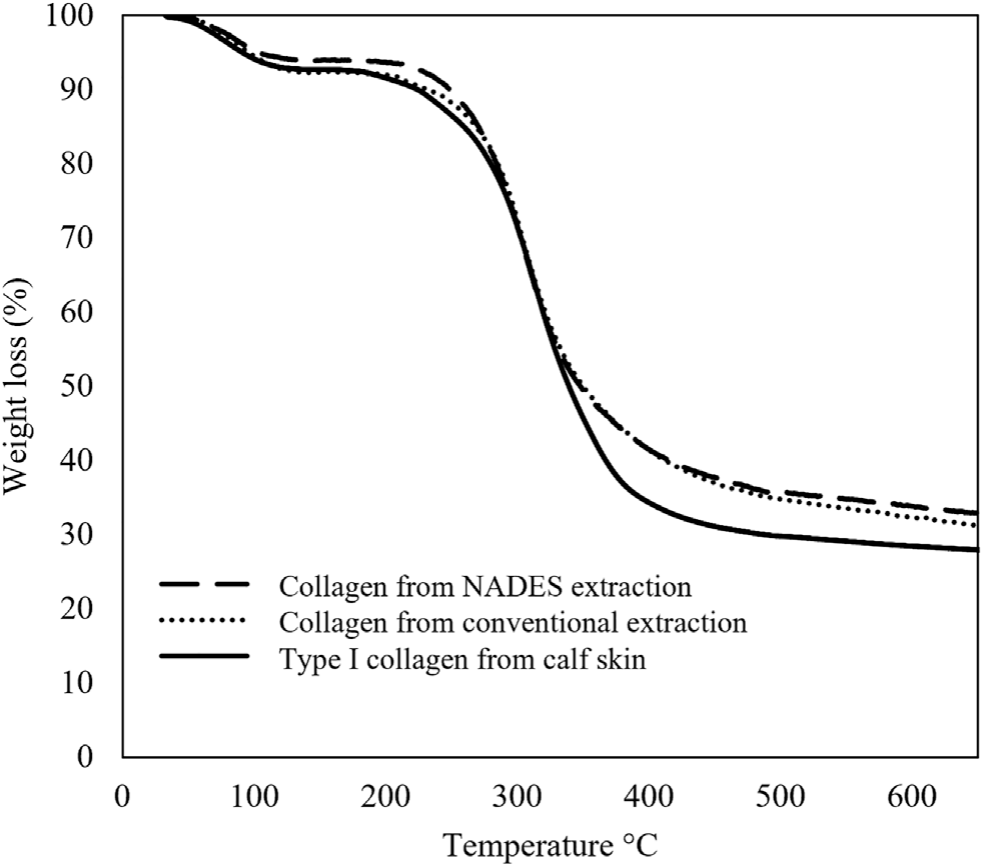
TGA data of the collagen extracts obtained from blue shark skins with conventional solvent and NADES and collagen standard from calf skin.

To confirm the purity of the collagen extracts, TGA was performed. Figure 3 compares the thermal behavior of the obtained samples with a commercial standard. TGA allows the analysis of different thermal decomposition profiles between samples, indicating the presence of potential contaminants.

The thermograms shown in Figure 3 present similar profiles, with the final mass percentage tending to the same values (30%). TGA curves present weight loss in the range from room temperature to 150°C due to water evaporation and between 200 and 500°C associated with the decomposition of collagen. The slight differences may be due to marginally different moisture content between samples. Therefore, these results suggest that the collagen extracts obtained in this work have high purity.

### 3.3 FTIR

**FIGURE 4:**
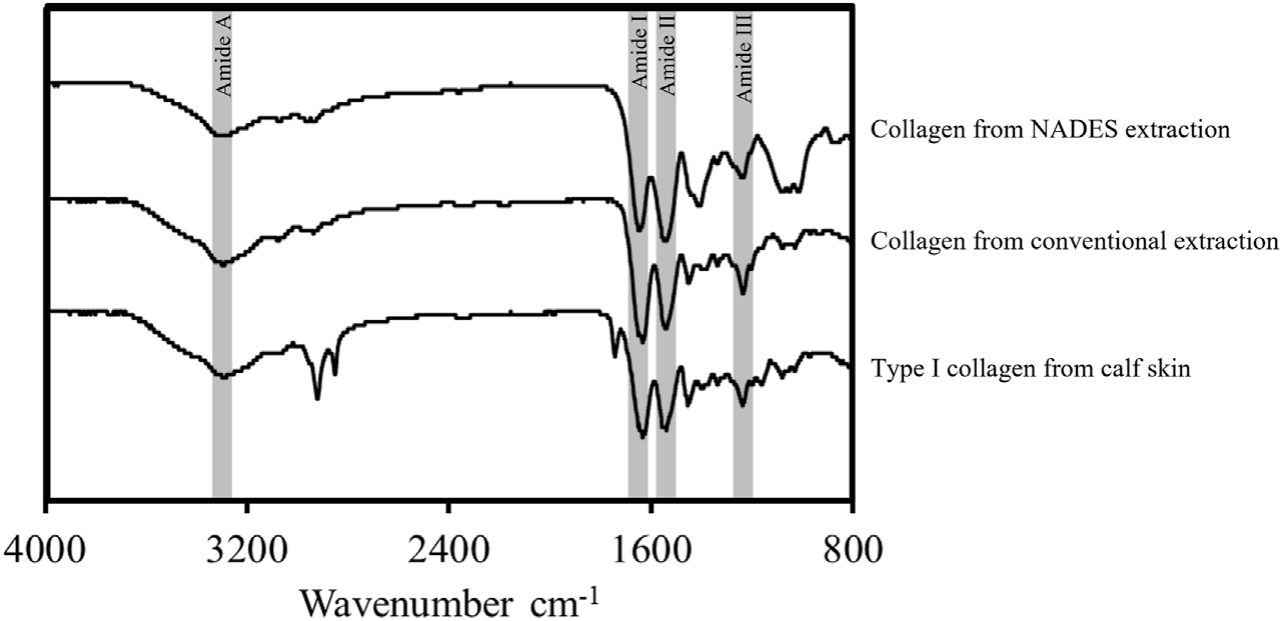
FTIR spectra of the collagen extracts obtained from blue shark skins with conventional solvent and NADES and collagen standard from calf skin, exhibiting the main vibrations of collagen molecular organization, amide A, amide I, amide II, and amide III.

The FTIR spectra of both collagen extracts from blue shark skin and commercial type I collagen from calf skin are exhibited in Figure 4.

Despite the different extraction conditions and collagen sources, the overall spectra profiles presented in Figure 4 are similar, suggesting that all samples show comparable structures and chemical compositions. Typical bands of type I collagen’s molecular chain, corresponding to amide A, amide I, amide II, and amide III, were observed (Silva et al., 2016; Elango et al., 2018; Jafari et al., 2020). The broad band of amide A, observed at 3000–3500 cm^−1^, is typical for the presence of N-H stretching coupled with hydrogen bonds. The peak for amide I, from the stretching vibrations of the carbonyl groups (C=O) in proteins, is observed at 1634 cm^−1^. The presence of amide II, attributed to N-H bending coupled with C-N stretching, is observed from the peak at 1550 cm^−1^. Finally, the peak of the amide III group from the N-H bending was identified at 1239 cm^−1^. The FTIR analysis suggested that the extracts correspond to type I collagen as the commercial standard also analyzed. These results indicate that both extraction methods did not damage the functional groups present in the collagen triple helix.

### 3.4 Circular Dichroism

**FIGURE 5:**
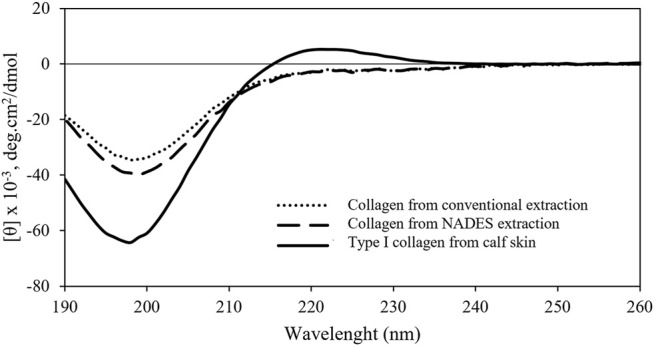
CD spectra of the collagen extracts obtained from blue shark skins with conventional solvent and NADES and type I collagen standard from calf skin.

To assess whether the extracted collagen is in its native triple helical structure, the CD spectra of the different extracts were obtained and compared with the commercial type I collagen standard. This spectroscopy tool assesses if type I collagen has a well-defined CD transition with a positive peak around 222 nm, representing a triple helix conformation (Carvalho et al., 2018; Jafari et al., 2020). The results of the CD spectroscopy analysis are shown in Figure 5.

A negative peak of the extracted collagens is close to the negative peak of collagen standard in the 199 nm region. However, unlike the native collagen sample, none of the extracted collagens presented a positive peak in the 222 nm region, characteristic of the *a* helix conformation. These results suggest that although the collagen extracts appear to have a rather complex secondary structure, at least partial protein denaturation occurred upon extraction. This could be due to the presence of dilute citric acid that could dissociate intermolecular interactions of collagen triple helix (Carvalho et al., 2018). These results suggest that the conventional and the proposed NADES methodologies do not ensure the total structural integrity of the biopolymer. However, the CD spectra similarity of both extracts suggests that the higher temperature of NADES extraction methodology is not responsible for the protein denaturation. Nevertheless, this has been a struggle found in different works of collagen extraction from other marine sources that reported similar spectra profiles, containing only a negative peak around 197–203 nm (Ikoma et al., 2003; Silva et al., 2016; Carvalho et al., 2018).

### 3.5 Hg Quantification

Several works have reported the bioaccumulation of Hg in blue shark tissues (Branco et al., 2004; Branco et al., 2007; Escobar-Sánchez et al., 2011). The presence of this contaminant can restrain the recovery of collagen from blue shark wastes for the development of novel biomedical products. Various international governing bodies such as the U.S. Pharmacopeia, European Commission or the U.S. Food and Drug Administration impose specific and strict regulations with limits for the elemental impurities content in products for biomedical, cosmetic, or food purposes (Barin et al., 2016; United States Phamacopeia - USP, 2016; CBER, 2022). The content of Hg impurities in shark skins and collagen extract was determined by atomic absorption spectrophotometry.

Table 2 presents the results of Hg quantification in the raw material and collagen extract obtained with NADES.

**TABLE 2:** Hg content of shark skins and collagen extract from NADES extraction.

Sample	Hg content (mg/kg dry weight)
Shark skins	1.64 (±0.48)
Collagen extract	< Detection limit (0.01 mg/kg)

The blue shark wastes used for collagen recovery in this work presented a contamination of 1.64 (±0.48) mg Hg/kg skins dry weight. However, Table 2 shows that the Hg content in the collagen extract obtained with the NADES extraction method was below the equipment detection and regulatory limits. These results suggest that the new methodology for collagen recovery presented in this work prevents the concentration of Hg impurities from raw material in the final extract.

### 3.6 Biocompatibility

#### 3.6.1 Cytotoxicity

**FIGURE 6:**
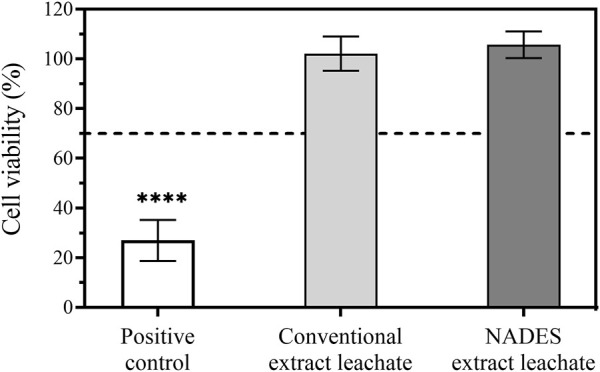
Cytotoxicity assay using MTS reagent: leached extracts were incubated in NCTC clone 929 cell line for 24 h at 37°C and 5% CO2 humidified atmosphere (mean ± SD, *n* = 3). Statistically significant differences comparing all conditions are indicated by **** (*p* < 0.0001).

To evaluate the potential of the extracted collagen for biomedical skin applications, the cytotoxicity on a mouse fibroblasts cell line was assessed (Figure 6). Due to the neutral pH of the cell culture medium, the extracted acid-soluble collagen does not solubilize, hindering the direct and homogeneous contact of the extract with the cells. So, the cytotoxicity of any leachables that can migrate from the extract to the skin cells was evaluated following the ISO 10993–5 methodology. This approach helps determine the biological reactivity of any substances that can be released from a medical device or material during clinical use and demonstrate the hazard potential of the product.

As shown in Figure 6, none of the leachates showed percentages of cytotoxicity below the standard threshold of 70%. The results show that possible leachable chemicals from the collagen extracts obtained with our novel methodology are noncytotoxic.

## 4 Conclusion

This work successfully extracted marine collagen from blue shark skins, a very abundant waste resulting from the fish industry. Collagen was obtained through a new sustainable, faster, and more straightforward extraction approach using NADES composed of citric acid:xylitol:water when compared with the conventionally used extraction. In fact, the procedure was effective without pre-treating raw materials, greatly reducing the extraction time from 96 to 1 h and, therefore, the associated processing costs. Additionally, the proposed methodology resulted in a 2.5 times improvement in extraction yield when compared to the traditional procedure, resulting in the isolation of more than 21% of the protein content of the blue shark skin studied in this work. The new operating conditions allowed to obtain collagen with similar properties in terms of purity, molecular weight, chemical composition, and cytotoxicity when compared to the collagen obtained by the conventional methodology. Overall, the obtained results provide essential data on the industry-favorable extraction process parameters, opening the possibility of a simple, quick, cost-effective, and environmentally sustainable process to obtain blue shark skin-derived collagen as a promising material for topical biomedical applications.

## Data Availability

The original contributions presented in the study are included in the article/Supplementary Material, further inquiries can be directed to the corresponding author.
