# Supplementary material for: Extraction of Biocompatible Collagen From Blue Shark Skins Through the Conventional Extraction Process Intensification Using Natural Deep Eutectic Solvents
Source: Front Chem. 2022 Jun 16;10:937036. doi: 10.3389/fchem.2022.937036 (PMC9243641; doi:10.3389/fchem.2022.937036)
Supplement: Supplementary file 1 [file Image1.pdf]

Supplementary material

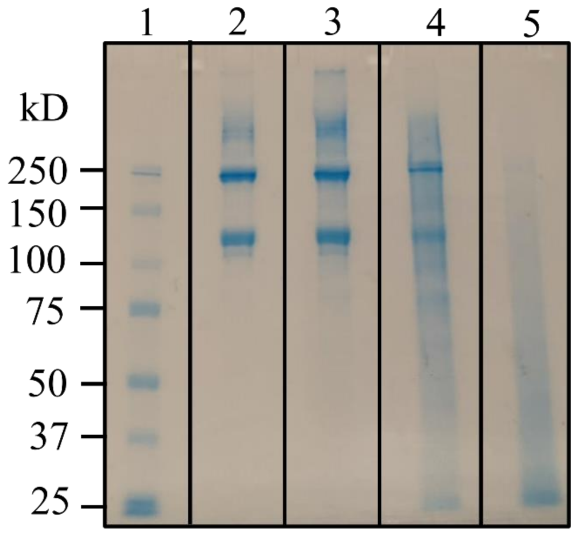

Figure S.1. Electrophoretic profile of molecular weight standards (1) and protein rich extract from NADES extraction performed at 40 °C (2), 60 °C (3), 80 °C (4) and 100 °C (5).
